# MRF-Net: A multi-branch residual fusion network for fast and accurate whole-brain MRI segmentation

**DOI:** 10.3389/fnins.2022.940381

**Published:** 2022-09-12

**Authors:** Chong Wei, Yanwu Yang, Xutao Guo, Chenfei Ye, Haiyan Lv, Yang Xiang, Ting Ma

**Affiliations:** ^1^Institute of Electronical and Information Engineering, Harbin Institute of Technology, Shenzhen, China; ^2^Peng Cheng Laboratory, Shenzhen, China; ^3^International Research Institute for Artificial Intelligence, Harbin Institute of Technology, Shenzhen, China; ^4^MindsGo Co., Ltd., Shenzhen, China; ^5^Advanced Innovation Center for Human Brain Protection, Capital Medical University, Beijing, China; ^6^National Clinical Research Center for Geriatric Disorders, Xuanwu Hospital Capital Medical University, Beijing, China

**Keywords:** deep learning, whole-brain segmentation, residual error fusion module, multi-branches cross-attention module, MRF-Net

## Abstract

Whole-brain segmentation from T1-weighted magnetic resonance imaging (MRI) is an essential prerequisite for brain structural analysis, e.g., locating morphometric changes for brain aging analysis. Traditional neuroimaging analysis pipelines are implemented based on registration methods, which involve time-consuming optimization steps. Recent related deep learning methods speed up the segmentation pipeline but are limited to distinguishing fuzzy boundaries, especially encountering the multi-grained whole-brain segmentation task, where there exists high variability in size and shape among various anatomical regions. In this article, we propose a deep learning-based network, termed Multi-branch Residual Fusion Network, for the whole brain segmentation, which is capable of segmenting the whole brain into 136 parcels in seconds, outperforming the existing state-of-the-art networks. To tackle the multi-grained regions, the multi-branch cross-attention module (MCAM) is proposed to relate and aggregate the dependencies among multi-grained contextual information. Moreover, we propose a residual error fusion module (REFM) to improve the network's representations fuzzy boundaries. Evaluations of two datasets demonstrate the reliability and generalization ability of our method for the whole brain segmentation, indicating that our method represents a rapid and efficient segmentation tool for neuroimage analysis.

## 1. Introduction

Neuroanatomy segmentation plays a key role in the analysis of the development of the neonatal brain, and the diagnosis of neurodegenerative diseases. For instance, segmentation is carried out on the brain for reaching volume, thickness, and morphological measurements. The brain MRI scans are usually segmented automatically by warping a manually annotated atlas to the target by a well-established pipeline such as FreeSurfer (Fischl et al., 2002) and FSL (Woolrich et al., 2004). Several steps are involved for extensive numerical optimization including image transformation, careful fine-tuning of parameters, smoothing, and even non-linear registration. Especially, the estimation of the 3D deformation field for non-linear registration is computationally intense and suffers from long runtime. The efficiency of this type of segmentation algorithm is extremely low, and it is not practical for large-scale data processing. Therefore, there is an urgent need for fast and accurate brain segmentation algorithms, so that medical research based on large-scale brain structure segmentation becomes feasible. It is challenging to develop fast and accurate whole-brain segmentation algorithms, due to the complex 3D brain structure, high-dimensional neuroimaging data, spatial dependency between slices, and numerous and imbalanced labels.

Recently increasing deep learning algorithms have been proposed for medical image semantic segmentation, e.g. brain lesion location. The CNN based methods provide insight into efficiently and effectively processing the whole brain in an end-to-end manner, which is feasible to segment the whole brain into parcels at the minute-level, at the same time surpassing conventional atlas-based methods in terms of segmentation accuracy. For instance, Roy et al. (2019) proposed the QuickNAT framework based on a deep fully convolution neural network that processes the 3D MRI T1 brain scans in seconds, which greatly improves the efficiency of segmentation and makes large-scale segmentation tasks feasible. FastSurfer (Henschel et al., 2020) introduced a competition block and included a wider context within a slice to improve spatial representations. However, these studies are still limited in dealing with multi-grained parcels and complex boundaries.

In this article, we propose a novel segmentation network architecture, Multi-branch Residual Fusion Network (MRF-Net), that could segment the whole brain into parcellations in seconds. Unlike most existing methods mentioned above, our network benefits from strengthening representations on multi-grained representations, especially on fuzzy boundaries. Our contributions can be summarized as:

- To tackle the limitation of the high variability in size and shape between regions, we propose a multi-branch cross-attention module (MCAM), which is able to explore locally distributed patterns with different respective fields, capturing and relating dependencies between representations in various scales.
- The fuzzy boundaries are improved by a residual error fusion module (REFM), that leverages the residual errors between slices to improve marginal representations for locating boundaries.
- Experimental evaluations on two datasets demonstrate that our method represents a rapid and efficient segmentation tool for morphometric neuroimage analysis, which achieves high reliability and generalization abilities in performance. The method achieves consistent improvements and surpasses the existing state-of-the-art methods with average dice scores of 81.70 and 86% in two datasets, respectively.

The rest of the article is organized as follows: We first present the related studies about the semantic segmentation on the whole brain tasks in Section 2. In Section 3, we introduce the studied two datasets and represent a detailed architecture of the proposed method, including the residual error fusion module (Section 3.4) and the multi-branch cross-attention module (Section 3.5). In Section 4, we conducted extensive experiments to evaluate the advantage of our method in four aspects including the segmentation performance in Section 4.1, the computational complexity in Section 4.2, ablation studies to evaluate the proposed modules in Section 4.3, and reliability performances with statistical analysis in Section 4.4. The conclusion is drawn in Section 5.

## 2. Related study

### 2.1. Traditional whole-brain segmentation

The whole-brain segmentation is always conducted by well-maintained MRI processing pipelines, such as FreeSurfer (Fischl et al., 2002), FSL (Woolrich et al., 2004), BrainSuite (Shattuck and Leahy, 2002), SPM (Penny et al., 2011), and ANTs (Avants et al., 2009). Such pipelines leverage atlas-based models for locating brain regions using registration approaches, leading to a cost of several hours of calculation time for a large number of graphics transformations.

### 2.2. Medical image segmentation

Deep learning methods have recently merged, that are able to achieve outstanding performances in image recognition (Ren et al., 2015; He et al., 2016; Grigorev et al., 2020) and text analysis (Vaswani et al., 2017; Devlin et al., 2018; Xu et al., 2020), and even inference in seconds. The extensive applications of deep learning in medical images have helped interpret medical scans, lesions, disorganized patterns, and morphological measurements (Topol, 2019; Yang et al., 2021a,b; Yin et al., 2021). Among them, UNet (Ronneberger et al., 2015) is a network that has made a breakthrough in the field of medical image segmentation. Subsequent studies have been focused on modification of the network architecture and achieves consistent improvements over UNet, such as UNet++, UNet3+, and nnU-Net (Isensee et al., 2018; Zhou et al., 2018; Yuan et al., 2019; Huang et al., 2020). In particular, UNet-like networks leverage a skip connection method to extract and retain both high-level and low-level semantic features, which improves the learned representations, at the same time reducing parameters by pruning unnecessary architectures. In addition, most forms of medical images are in 3D dimension, so 3D segmentation networks such as 3D-UNet and V-Net have been proposed by Çiçek et al. (2016), Liu et al. (2018), Milletari et al. (2016), Myronenko (2018), and Jiang et al. (2019) and achieved considerable results, outperforming 2D methods in some tasks. However, due to the complexity of computation of the 3D feature maps, the 3D methods are difficult to be applied in practice, especially for some tasks dealing with large-scale images. To reduce the computational cost, 2.5D segmentation methods have been proposed by Yu et al. (2019) by receiving several 2D slices as an input. These segmentation algorithms will not occupy a great number of computing resources while retaining local spatial information. Therefore, our study is implemented in this way to improve efficiency, which can be easily applied in practice.

### 2.3. Whole-brain segmentation

To date, there are some deep learning networks for whole-brain structure segmentation. The segmentation algorithm based on a 3D network requires high computing resources, resulting in slow computing speed (Huo et al., 2019), and requires a lot of preprocessing work. These preprocessing steps are error-prone, so it is not convenient for large-scale applications. SD-Net is the first to achieve end-to-end full convolutional network segmentation, which could segment the brain into 27 classes, so its practicality is limited (Roy et al., 2017). QuickNAT (Roy et al., 2019) propose the fusion of three views based on SD-Net, which greatly improves the accuracy of whole-brain structure segmentation. Finally, the state-of-the-art CNN based whole-brain segmentation network is FastSurfer (Henschel et al., 2020), which has made more in-depth improvements based on QuickNAT. It proposes competitive dense blocks (CDB) to replace ordinary dense blocks and competitive connections instead of skip connections. Additionally, it can segment the brain into 95 classes, which greatly improves the practicality of FastSurfer in medical research.

## 3. Materials and methods

### 3.1. Datasets

In this article, we built two datasets for training, validation, and testing the performance of our proposed MRF-Net. Note that, all the sets are selected with matched age and gender. For evaluation, each dataset is split into a training set with 70% samples, a validation set with 15% samples, and a test set with 15% samples. Within both two datasets, healthy controls are included balanced with gender, age, and scanning parameters (i.e., scanners, field strength, and acquisition parameters).

**Johns Hopkins University (JHU)**. The JHU dataset was collected from the JHU brain atlas repository by Ye et al. (2018) and Wu et al. (2016) with 136 T1 brain magnetic resonance images (MRI) with ages ranging from 22 to 90. These images were divided into 136 parcels *via* the MRICloud platform (https://braingps.MRICloud.org) and corrected by clinicians manually. The data were acquired using the MPRAGE sequences at JHU using 3T Philips scanners with 1 mm isotropic resolution.

**The Alzheimer's Disease Neuroimaging Initiative (ADNI)**. The ADNI dataset was built based on the ADNI database (Mueller et al., 2005), containing 5074 T1 whole-brain MRI, each of which was segmented into 138 brain regions (On average, 2 parcels are missing for each data) by a validated method (MALPEM) (Ledig et al., 2018). The study was done in the BioMedIA group at Imperial College London, UK. We randomly collected 210 data of normal control in this study. T1-weighted (T1w) MR brain images were collected from the Alzheimer's Disease Neuroimaging Initiative (ADNI) including ADNI-1, ADNI-GO, and ADNI-2.

### 3.2. Methods overview

**Figure 1:**
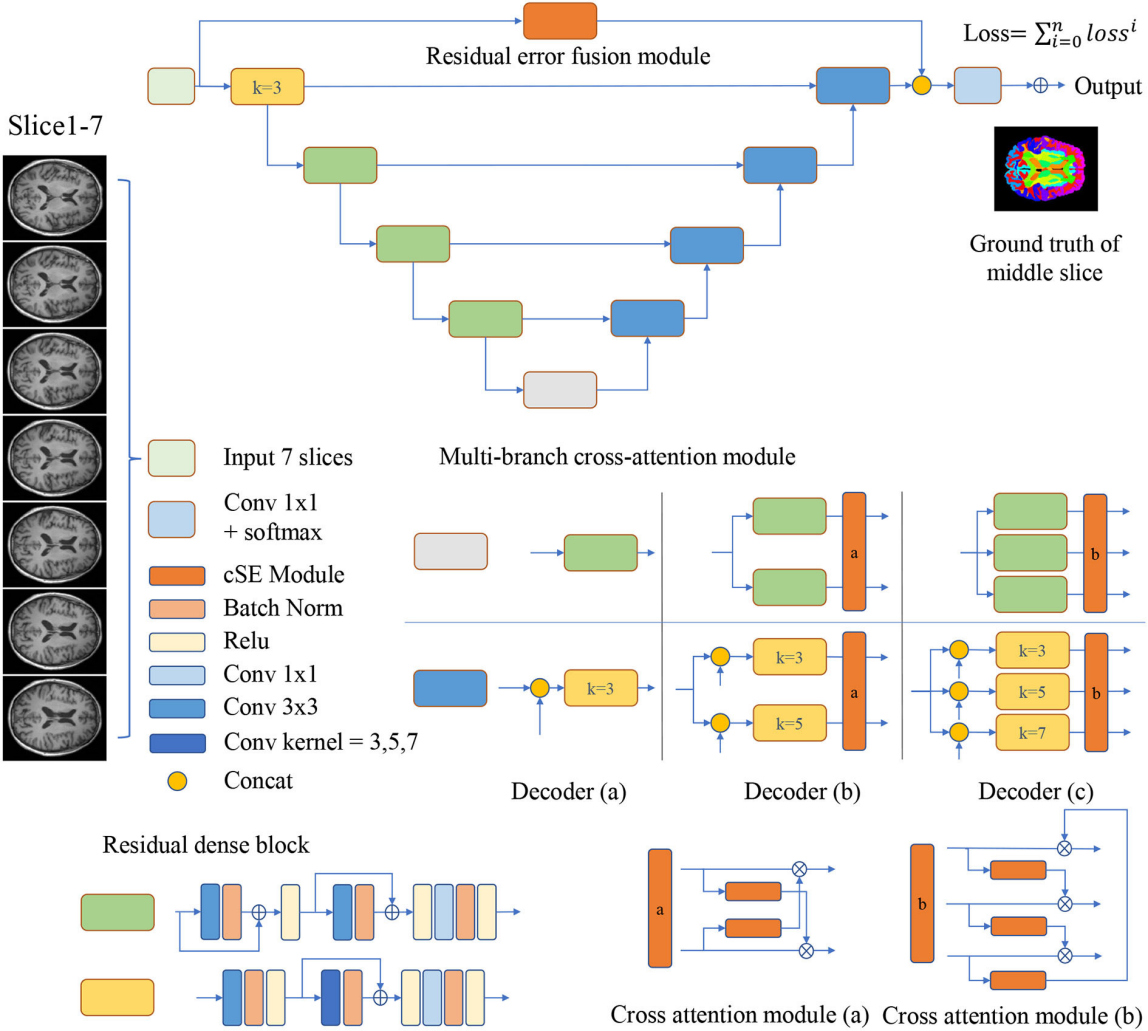
Multi-branch Residual Fusion Network (MRF-Net) structure. Operations are shown in different colors, with detailed information on the right side. For the multi-branch cross-attention module, a detailed structure diagram is displayed and represented by the decoder **(a–c)**, where k represents the size of the convolution kernel.

As is shown in Figure 1, the encoder-decoder structure is implemented as the basis for MRF-Net. For the 2.5D image segmentation, the input is obtained by dividing the raw image into blocks by every 7 slices along the axial or coronal plane, where the ground truth of the mediate slices is used as supervision.

### 3.3. Residual dense blocks

The encoders and decoders are constituted by residual dense blocks, where each residual dense block contains 3 convolution layers with 64 channels. The residual dense block is improved from the CDB of FastsurferCNN (Fischl et al., 2002). In each residual dense block, the short-distance residual connections are added to the convolution layer instead of maxout in CDB. And change all convolution kernel size to 3. The bottom feature is split into *N*∈{1, 2, 3} branches by a gray block.

### 3.4. Residual error fusion module

**Figure 2:**
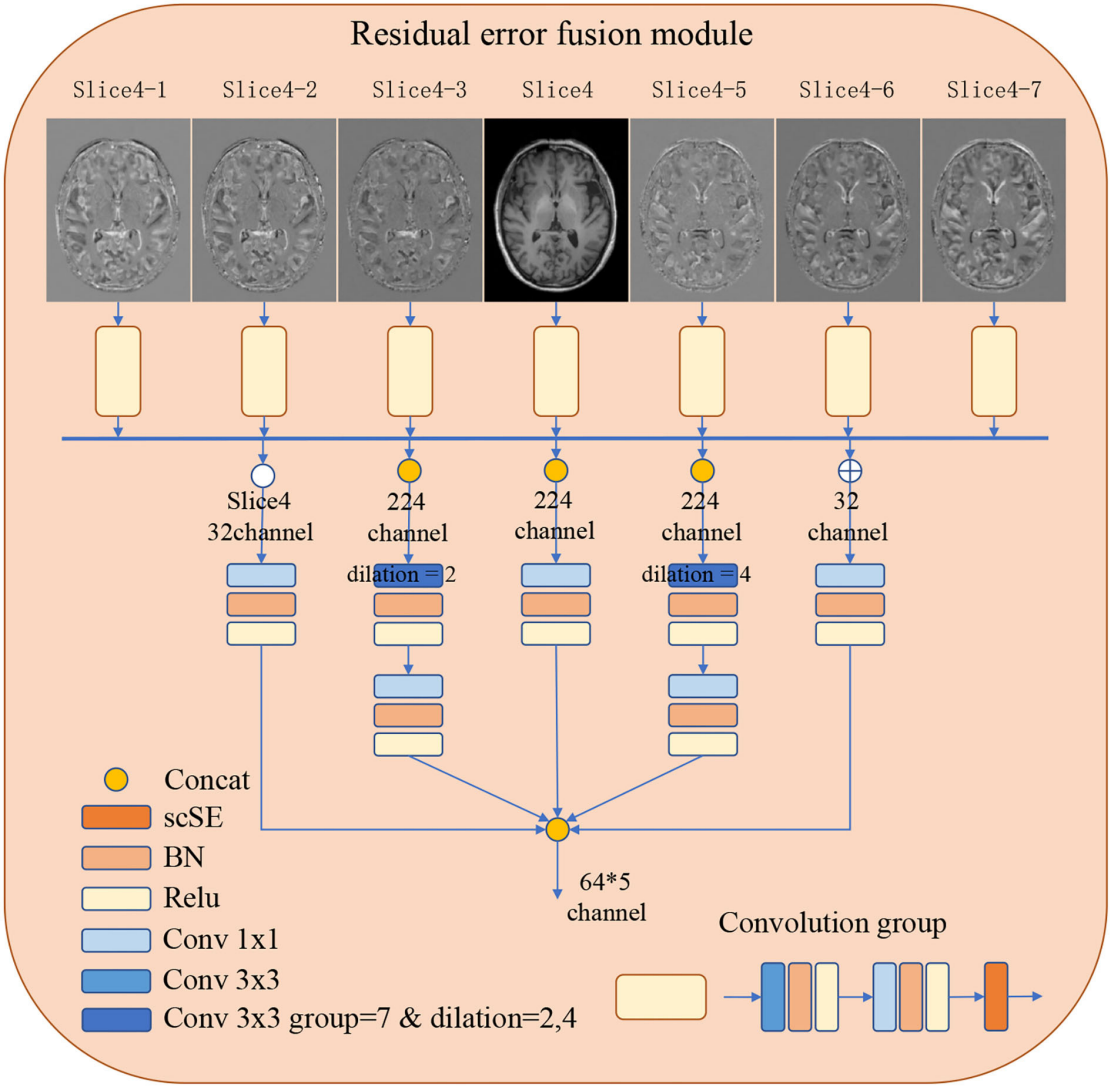
The detailed architecture of the Residual error fusion module (REFM). Operations are shown in different colors, with a detailed description on the left side. The output of the module is further cascaded to the classifier.

In order to improve the representations of the boundary details of each brain region, here, we calculate the difference between the input slices to provide local spatial boundary information. For 7-slice image input, 7 encoding branches are implemented, where the input of the middle branch is the fourth slice. Subsequently, the input of the remaining branches is the difference between the fourth slice and other slices. Each branch is constructed with two layers of convolution and a scSE (Roy et al., 2018) attention module. The scSE module contains a channel attention module and a spatial attention module. The obtained 7 feature maps further extract features through 5 operations, they are: (1) concatenation and convolution; (2) concatenation and group convolution (group = 7, dilation = 2); (3) cascade and group convolution (group = 7, dilation = 4); (4) concatenation and convolution; (5) Convolution operation on the middle slice. The feature maps of the five branches are cascaded to the classifier. The use of dilated convolution and group convolution is to expand the receptive field of the network without causing confusion of information. This module realizes the extraction of boundary detail features and multi-scale information. Specific definition is shown in Figure 2.

### 3.5. Multi-branch cross-attention module

The MCAM is designed with two or three branch decoders with different sizes of kernels embedded in order to locate multi-grained contextual information. These blocks are connected with a cross-attention module for mixing and synthesizing multi-grained information. In detail, the decoder (a) contains just one branch, the size of the convolution kernel in the middle of the convolution group is 3, which will concatenate the feature maps from the encoder, and decoder (a) is used to analyze the performance. The two branches of the decoder (b) contain residual dense blocks with kernel sizes of 3 and 5, respectively. The two branches will concatenate the features from the encoder, respectively, and then get two feature maps, and the cross-attention module will fuse the multi-scale features and upsample to get two feature maps and send them to the next convolution group. The three branches of the decoder (c) contain residual dense blocks with kernel sizes of 3, 5, and 7, respectively (Dou et al., 2020). The cross-attention module will fuse multi-scale features and upsample to obtain three feature maps, which will be sent to the next convolution group. The decoder achieves the highest segmentation accuracy.

The *N* branch's decoder is calculated by:


(1) $$\begin{array}{r} y_{i}=F_{i}\left(x_{i},skip\right) \end{array}$$

where *x*_*i*_ and *y*_*i*_ denote the *i*-th branch's input and output feature, respectively, function *F*_*i*_(*x*_*i*_, *skip*) denotes the *i*-th branch's RDB, and *skip* denotes the skip feature from the encoder.

Our proposed cross-attention module contains two or three identical channel attention modules. The original channel attention is proposed by Roy et al. (2018), and we use it in a new way. The channel attention is calculated by:


(2) $$\begin{array}{c} g_{c}(x_{c})=\frac{1}{H\times W}\sum_{h}^{H}\sum_{w}^{W}x_{c}(h,w) \end{array}$$

(3) $$\begin{array}{r} G_{i}\left(x_{i}\right)=\sigma \left(W_{1}Relu\left(W_{2}g\left(x_{i}\right)\right)\right) \end{array}$$

where *x*_*i*_ denotes the *i*-th branch's input feature, *H* and *W* denote the height and width of features, *g*_*c*_ denotes the *c* channel's average value, *W*_1_ and *W*_2_ denote two fully-connected layers, σ denotes the sigmoid layer, *G*_*i*_(*x*_*i*_) denotes the channel attention map of the *i*-th branch. It multiplies the channel attention map of the current branch with the next branch to promote multi-grained contextual information transforming among branches of different grains. The cross-attention module is defined as:


(4) $$\begin{array}{r} y_{i}=x_{i}⊙G_{\left(i+1\right)\%N}\left(x_{\left(i+1\right)\%N}\right) \end{array}$$

where % denotes the remainder operation, ⊙ denotes the channel-wise multiplication. The cross-attention modules can be added behind multi-branch decoders. Two different cross-attention modules are designed for the decoder (b,c), where Figure 1 demonstrates the details.

### 3.6. Implementation details

Our experiments are conducted in Pytorch with one NVIDIA Tesla GV100-32G. Adam is used to optimize the parameters of the networks. The batch size in the training phase is set as 8. The average value of dice loss and cross-entropy loss is used for training. We set the initial learning rate to 0.001, and multiply the learning rate by 0.1 every 10 epochs. Our loss function is shown as:


(5) $$\begin{array}{r} Loss\left(X,Y\right)=1-\frac{2\times XY}{X+Y}-XlogY \end{array}$$

The evaluation index adopts the Dice coefficient and average Hausdorff distance, and the calculation formula of the Dice coefficient is:


(6) $$\begin{array}{r} Dice\left(X,Y\right)=\frac{2\times XY}{X+Y} \end{array}$$

The formula for calculating the average Hausdorff distance is:


(7) $$\begin{array}{r} HD\left(X,Y\right)=\frac{1}{|X|}\underset{x\in X}{\sum }min_{y\in Y}d\left(x,y\right)+\frac{1}{|Y|}\underset{y\in Y}{\sum }min_{x\in X}d\left(y,x\right) \end{array}$$

where Y and X denote the predicted probabilities and the ground truths of the image, respectively. |*X*| and |*Y*| represent the number of voxels in the binary label maps of ground truth X and prediction Y.

## 4. Results

### 4.1. Comparison with state-of-the-art methods

Our proposed MRF-Net was compared with the state-of-the-art FastSurferCNN (Henschel et al., 2020), UNet++ (Zhou et al., 2018), and UNet 3+ (Huang et al., 2020) for whole-brain structure segmentation. The dice scores and average Hausdorff distance of 3D results segmented from the validation set and the test set are reported in Tables 1, 2, respectively, where the best results are shown in bold. The experimental results show that our proposed MRF-Net with the decoder (c) outperforms other methods in terms of the dice score with 81.70%/86.00% in the JHU/ADNI dataset, respectively. In addition, the evaluation results on the test set with unseen data are comparable to the validation set, indicating that the methods achieve outstanding generalization ability for the whole brain segmentation. The advanced deep learning tools bring new insight for neuroimaging processing tools with fast and accurate performance.

**Table 1:** The average Dice scores for 4 comparative networks on the axial segmentation results.

**Dice**	**JHU**		**ADNI**	
	**Validation**	**Test**	**Validation**	**Test**
FastSurfer	79.83 ± 3.08	80.29 ± 4.22	84.64 ± 4.50	84.72 ± 1.70
Unet++	80.08 ± 3.10	80.12 ± 5.68	85.26 ± 4.74	85.70 ± 2.07
Unet+++	80.57 ± 3.01	81.49 ± 3.11	85.81 ± 3.87	85.35 ± 2.20
MRF-Net + decoder(b) (ours)	81.10 ± 2.99	81.36 ± 2.95	86.35 ± 3.76	85.84 ± 1.44
MRF-Net + decoder(c) (ours)	**81.36 ± 3.09**	**81.70 ± 1.57**	**86.45 ± 4.34**	**86.00 ± 0.83**

The names of our networks are shown in the form of MRF-Net + decoder. The best results are shown in bold.

**Table 2:** The hausdorff distance scores for 4 comparative networks on the axial segmentation results.

**Hausdorff**	**JHU**		**ADNI**	
	**Validation**	**Test**	**Validation**	**Test**
FastSurfer	0.299 ± 0.046	0.216 ± 0.033	0.266 ± 0.239	0.580 ± 0.706
Unet++	0.292 ± 0.053	0.263 ± 0.215	0.427 ± 0.422	0.728 ± 0.707
Unet+++	0.269 ± 0.051	**0.169 ± 0.034**	0.399 ± 0.315	0.836 ± 0.903
MRF-Net + decoder(b) (ours)	0.272 ± 0.045	0.229 ± 0.036	0.203 ± 0.102	0.500 ± 0.443
MRF-Net + decoder(c) (ours)	**0.261 ± 0.045**	0.173 ± 0.023	**0.201 ± 0.170**	**0.404 ± 0.100**

The best results are shown in bold.

Moreover, we carried out ablation studies on the different planes. To note that, the 2.5D segmentation methods are limited to be conducted on the saggital planes, where the spatial information of the left and right hemispheres are missed and convolutions fail to distinguish the position information. As is shown in Tables 3, 4, consistent improvements are achieved on both the axial and the coronal planes, and results on the coronal plane are better than those on the axial plane except for the MRF-Net with decoder (c) on the JHU datasets. Overall, our proposed method is robust in segmenting the whole brain along both two planes.

**Table 3:** Average dice score across the axial and coronal segmentation results.

**Hausdorff**	**JHU**		**ADNI**	
	**Axial**	**Coronal**	**Axial**	**Coronal**
FastSurfer	79.83 ± 3.08	80.04 ± 3.09	84.64 ± 4.50	85.40 ± 2.87
Unet++	80.08 ± 3.10	80.28 ± 3.11	85.26 ± 4.74	86.15 ± 1.94
Unet+++	80.57 ± 3.01	80.76 ± 3.04	85.81 ± 3.87	86.26 ± 3.67
MRF-Net + decoder(b) (ours)	**81.10 ± 2.99**	**81.20 ± 3.01**	**86.35 ± 3.76**	**86.42 ± 2.02**
MRF-Net + decoder(c) (ours)	**81.36 ± 3.09**	**81.26 ± 3.04**	**86.45 ± 4.34**	**86.68 ± 1.92**

The best results are shown in bold.

**Table 4:** Average hausdorff distance score across the axial and coronal segmentation results.

**Hausdorff**	**JHU**		**ADNI**	
	**Axial**	**Coronal**	**Axial**	**Coronal**
FastSurfer	0.299 ± 0.046	0.287 ± 0.048	0.266 ± 0.239	0.219 ± 0.053
Unet++	0.292 ± 0.053	0.274 ± 0.052	0.427 ± 0.422	0.200 ± 0.051
Unet+++	**0.269 ± 0.051**	**0.257 ± 0.049**	0.399 ± 0.315	0.197 ± 0.061
MRF-Net + decoder(b) (ours)	0.272 ± 0.045	0.259 ± 0.047	**0.203 ± 0.102**	**0.189 ± 0.043**
MRF-Net + decoder(c) (ours)	**0.261 ± 0.045**	**0.255 ± 0.050**	**0.201 ± 0.170**	**0.183 ± 0.040**

The best results are shown in bold.

**Figure 3:**
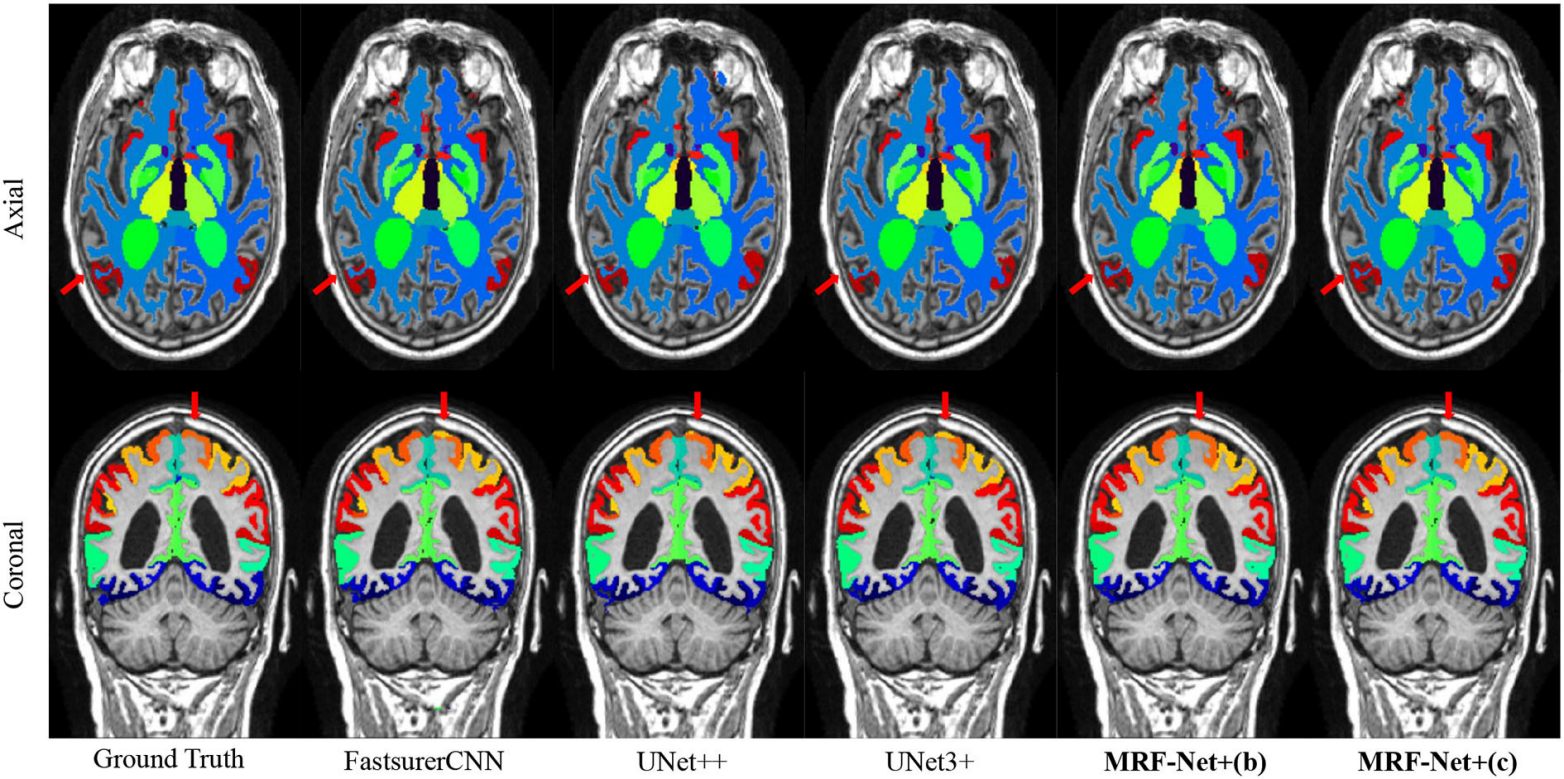
Segmentation results on the ADNI dataset. The axial plane and coronal plane results are shown, respectively. The red arrow highlights regions where the MRF-Net improved.

Figure 3 further displays the detailed results of the segmentation results. From the visualization view of the results, it can be observed that our proposed MRF-Net improves the segmentation accuracy in the boundary and optimizes the fuzzy margins compared with other methods.

### 4.2. FLOPs and parameters

**Figure 4:**
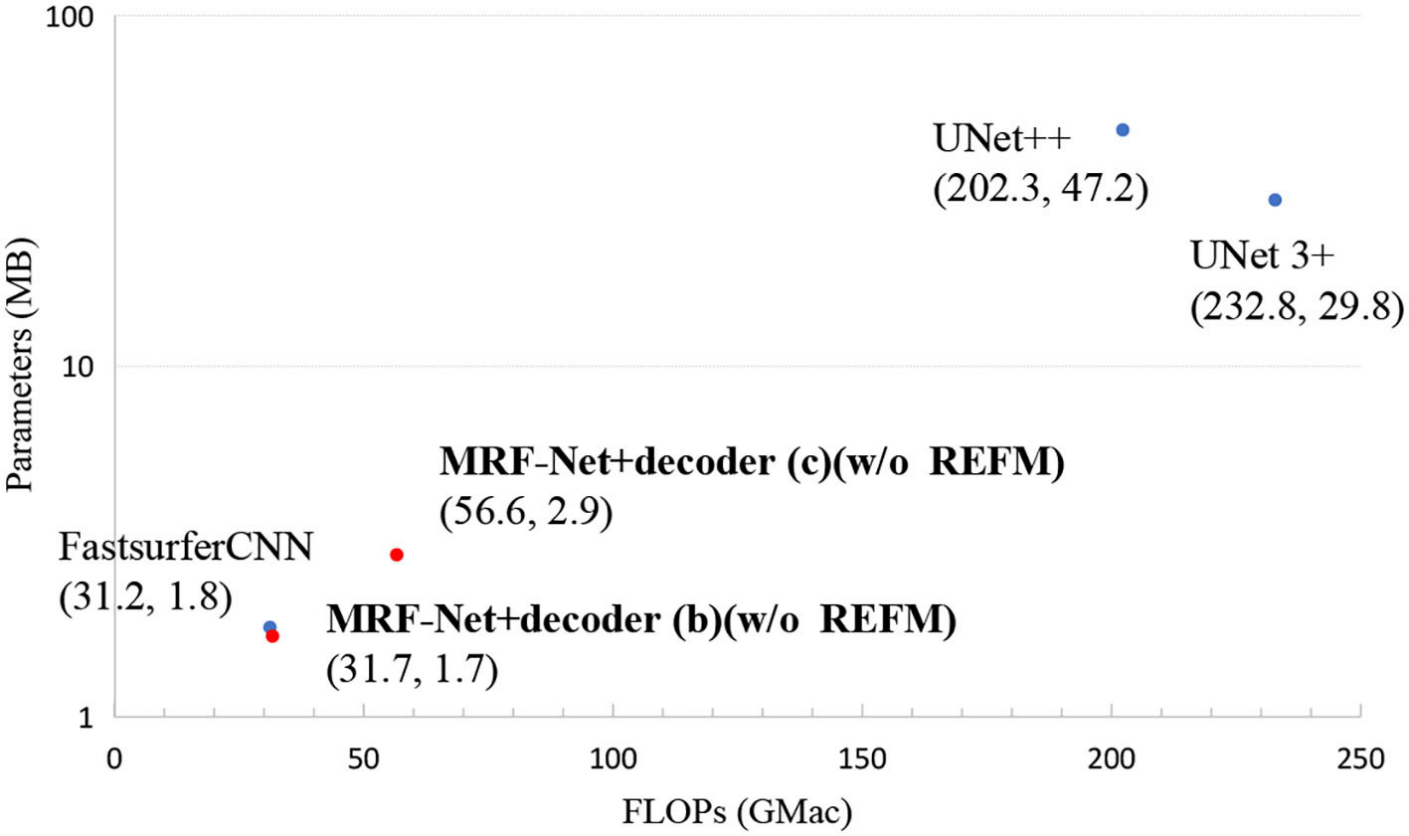
The FLOPs and total parameters of all networks. Scatter plots show FLOPs and parameters. Each point represents a network with a detailed name and location annotated next to the corresponding point. The results of our networks are bolded.

In order to show the practicability of MRF-Net, we report the FLOPs and total parameters of all the networks. Figure 4 shows that our network MRF-Net+decoder (b) (w/o REFM) surpasses the state-of-the-art networks in terms of total parameters while maintaining a high speed. By reference to Tables 1, 2, it can be observed that the MRF-Net with decoder (b) achieves better performance than FastSurfer with comparable parameters. A more complex decoder (c) can achieve higher accuracy, but the speed has slowed down, and the total amount of parameters has increased. Overall, the two kinds of decoder—decoder (b) and decoder (c) provides an alternative way for clinic applications. The MRF-Net with decoder (b) is more feasible for clinical applications that are limited in computational resources and speed. The MRF-Net with decoder (c) provides a more accurate tool with lower speed to some extent.

### 4.3. Ablation study

We demonstrated the effectiveness of residual dense blocks, REFM and MCAM through ablation studies. First, we set up an ablation experiment for residual dense blocks, the performance of 4 networks is reported to study the influence of the backbone on whole-brain segmentation. The first two networks adopt the same decoder (a) in Figure 1, and the Resnet50 (He et al., 2016), VGG16 (Simonyan and Zisserman, 2014) as encoders, respectively. Specifically, the connection method of resnet50+decoder(a) is: the decoder (a) concatenates the output feature maps of the start layer, layer1, and layer2 of resnet50 by skip connection, and finally upsampling twice to get the original size. The connection method of vgg16+decoder(a) is: the decoder (a) concatenates the output feature maps of every layer of vgg16 by skip connection. It can be seen from Table 5 that the complex backbones do not perform better. And our MRF-Net+decoder (a) (w/o REFM) performs better than FastsurferCNN. Too much low-level semantic information is lost in Resnet50 (He et al., 2016) and VGG16 (Simonyan and Zisserman, 2014) because of their simple low-level encoding layers, but our residual dense blocks could encode low-level semantic information effectively so that the decoder can restore more detailed information. In this experiment, we conclude that the complex high-level semantic information encoding cannot greatly improve the accuracy of the whole-brain segmentation. In terms of this, the decoding part and low-level semantic information are focused on the next experiments.

**Table 5:** The Dice scores of 4 networks.

**Dice**	**JHU**	**ADNI**
Resnet50+decoder (a)	79.66 ± 3.08	83.05 ± 4.56
VGG16+decoder (a)	79.43 ± 2.94	82.45 ± 4.94
FastsurferCNN	79.83 ± 3.08	84.64 ± 4.50
**MRF-Net +decoder (a) (w/o REFM)**	**80.32**±3.20	**85.39**±4.17

The last of them are designed by us, and they are represented by encoder + decoder. The axial segmentation results of the JHU dataset and ADNI dataset are reported. The best results are shown in bold.

**Figure 5:**
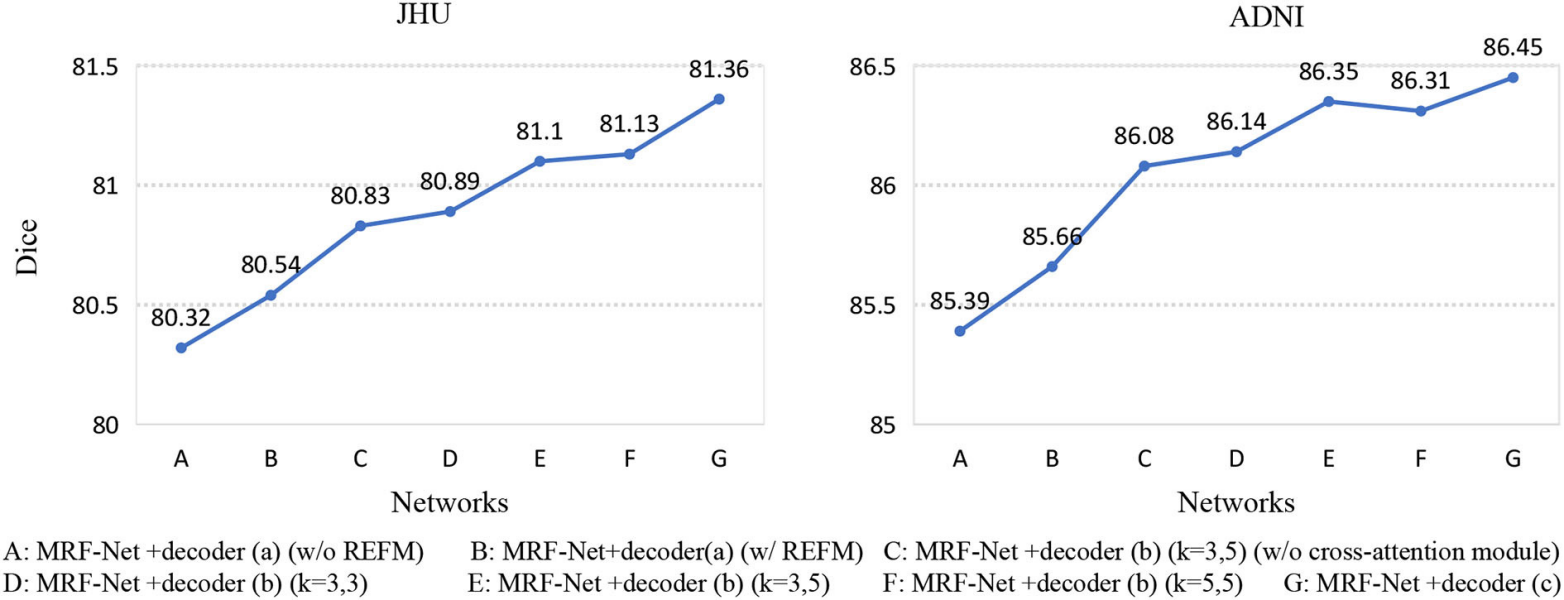
Seven networks are designed to verify REFM and MCAM. (w/o REFM) denotes that the decoder does not contain the Residual error fusion module. We add different decoders to the same encoder, and each network is represented by a letter, and their detailed correspondence is listed on the bottom side of the figure. Among them, (w/o cross-attention module) denotes that the decoder does not contain the cross-attention module, and (*k* = 3, 5) denotes that the size of the convolution kernel of the middle layer of the different branches in the decoder is (3, 3) and (5, 5), respectively.

Second, we set up an ablation experiment for REFM and MCAM, Figure 5 shows the Dice scores of 7 different networks on the two datasets. The 7 networks are represented as encoder+decoder, the details of which are shown in Figure 5. Network A and network B show the effectiveness of REFM, with additional input boundary information, which can effectively help the network segment fuzzy boundaries among brain regions. Network C can significantly improve the performance of whole-brain segmentation compared to the single-branch network B. The performance of network E is better than that of network C, so the cross-attention module helps promote the context transferring between two branches. Additionally, it can be observed that the increasing trend from network D to network E is obvious, but the Dice scores of network E and network F are similar. Therefore, when the kernels of the middle layer of two branches are different, the multi-grained contextual information could improve the segmentation performance with fewer parameters. Finally, according to the conclusion above, the decoder (c) is designed, which achieves the highest Dice score.

### 4.4. Reliability analysis

**Figure 6:**
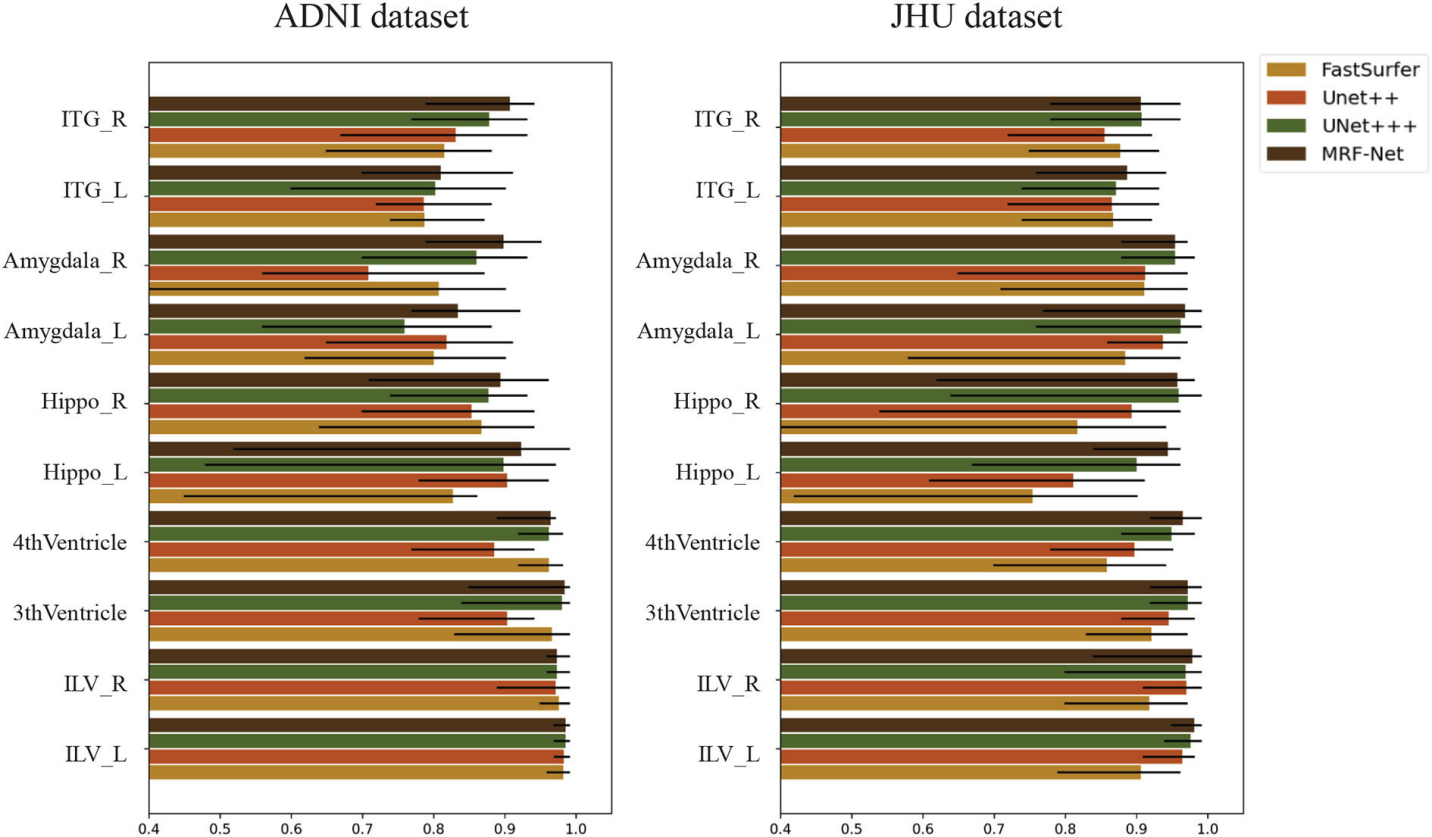
The intraclass correlation coefficient in terms of segmented structural volume based on four methods on 10 structures. The error bars represent the upper and lower bound of the ICC with a significance level of α = 0.05. ITG, Inferior Temporal Gyrus; Hippo, Hippocampus; ILV, Inferior Lateral Ventricle; L, Left hemisphere; R, Right hemisphere.

We evaluate the intraclass correlation coefficient (ICC) on the test set of both two datasets at the group-level. Figure 6 shows the intraclass correlation coefficient value on 10 structures for the four methods including Fastsurfer, Unet++, Unet+++, and MRF-Net, with the upper and lower bound at significance level α = 0.05 shown in black. The ICC value in the range of 0-1 reflects the reliability of the methods, and a bigger value indicates a better performance. It is shown that our proposed MRF-Net outperforms the other three methods over these structures on both datasets. High reliability performances (ICC > 0.975) are achieved on the left inferior lateral ventricle and the right inferior lateral ventricle in both datasets. In addition, the consistent improvements demonstrate that the proposed method achieves more robust performances than other state-of-the-art methods, although there are differences in reliability performance between the two datasets, especially in the left amygdala, which might be caused by the applied templates in the two tasks. In addition, the confidence intervals of the MRF-Net are smaller than other methods, indicating that our proposed method achieves a better segmentation consistency.

Moreover, the group-level and individual-level ICC and Pearson correlation coefficient (PCC) are also compared, which are summarized in Tables 6, 7. The individual-level comparison is obtained by measuring the corresponding score among the segmentation volume and the ground truth for each image, and the mean and the SD are calculated across the test subjects. The group-level comparison is conducted by measuring across the whole dataset for each brain parcel. The displayed scores are averaged across the whole brain, which is corresponded to a previous study by Henschel et al. (2020). In addition, to show the improvement of the reliability, the performance improvements over the FastSurfer method are shown with statistical scores, where pairwise tests are implemented on the improvements to evaluate significance. Our proposed MRF-Net exhibits high reliability on the ADNI dataset at both individual-level and group-level. For each image, the reprehensibility is the highest with the ICC score of 0.9977, and a significant improvement (*p* = 0.023) is achieved compared with the FastSurfer. Moreover, the PCC scores with MRF-Net are the highest among the four methods on both two datasets, indicating that the evaluated results of MRF-Net are more closely to the ground truth. Consistent significant improvements are obtained in both datasets (*p* < 0.001 and 0.033 for the JHU and the ADNI datasets, respectively).

**Table 6:** Estimations of volume on intraclass correlation coefficient (ICC) in terms of individual-level and group-level.

**Methods**	**JHU**			**ADNI**		
	**Individual PCC**	***p*-value**	**Group PCC**	**Individual PCC**	***p*-value**	**Group PCC**
FastSurfer	0.9678 ± 0.0476	-	0.812 ± 0.125	0.9953 ± 0.0043	-	0.881 ± 0.099
Unet++	0.9657 ± 0.0244	0.112	0.811 ± 0.107	0.9956 ± 0.0038	0.1370	0.894 ± 0.099
Unet+++	**0.9889 ± 0.1087**	<0.001***	**0.831 ± 0.117**	0.9957 ± 0.0039	0.0680	0.908 ± 0.104
MRF-Net + decoder (c)	0.9697 ± 0.0187	0.248	0.814 ± 0.126	**0.9977 ± 0.0047**	0.023*	**0.939 ± 0.041**

The best results are shown in bold. *p value < 0.05; ***p value < 0.001.

**Table 7:** Estimations of volume on Pearson correlation coefficient (PCC) in terms of individual-level and group-level.

**Methods**	**JHU**			**ADNI**		
	**Individual PCC**	***p*-value**	**Group PCC**	**Individual PCC**	***p*-value**	**Group PCC**
FastSurfer	0.9769 ± 0.032	-	0.783 ± 0.115	0.9959 ± 0.0037	-	0.914 ± 0.064
Unet++	0.9698 ± 0.210	0.033*	0.782 ± 0.120	0.9961 ± 0.0034	0.209	0.921 ± 0.064
Unet+++	0.9917 ± 0.005	<0.001***	0.791 ± 0.118	0.9963 ± 0.0033	0.165	0.929 ± 0.085
MRF-Net + decoder (c)	**0.9935 ± 0.005**	<0.001***	**0.798 ± 0.125**	**0.9979 ± 0.0042**	0.033*	**0.938 ± 0.035**

The best results are shown in bold. *p value < 0.05; ***p value < 0.001.

## 5. Discussion

Our proposed brain segmentation network MRF-Net could segment T1 brain MRI into 136 parcels, and compared with other CNN based methods, the experiments demonstrated that the MRF-Net achieves outstanding performance in terms of accuracy and reliability. Compared with the traditional methods that cost several hours for segmentation, our network not only completes the segmentation within 1 min (on the GPU) but also requires a little complex preprocessing work. For example, atlas-based methods such as FreeSurfer and FSL (Fischl et al., 2002; Woolrich et al., 2004) implement complex registration algorithms for segmentation, resulting in numerous time in image transformations, and are also limited by parameter setting that plays an important role in image registration. Moreover, researchers need to be very familiar with the software or the tool, which costs much time for researchers to learn to use and explore the detailed parameter settings. Deep learning technology can mitigate these shortcomings. It does not require complex parameter settings and inference time and has a strong generalization. This kind of method is easy to be applied and suitable for whole-brain segmentation tasks.

The high variability in shape and structure among regions is not avoidable in the whole brain segmentation. This characteristic results in the fuzzy boundary, which cannot be easily distinguished even by doctors. In terms of this, our proposed REFM is proposed to help strengthen the network's learning of fuzzy boundary information by implementing the residual marginal image difference. The model takes the residual information between the 7-layer slices as inputs and aggregates residual differences among slices to enhance the spatial information of margins. Through the information fusion of the 7 branches, a rich feature map containing low-level semantic information is finally obtained. According to the ablation experiment, we can clearly see from the ablation study that compared with MRF-Net +decoder (a) (w/o REFM), the network B with REFM increased by approximately 0.2% Dice coefficient.

For the multi-grained problem of whole-brain segmentation, we found that existing networks such as FastSurfer and QuickNAT (Roy et al., 2019; Henschel et al., 2020). only increase the receptive field by increasing the scale of the convolution kernel, which is obviously rough for the multi-grained features of whole-brain MRI. In this way, the MCAM module is designed, which consists of multiple branches of convolution kernels of different sizes, which can help the network identify multi-grained features better. From the related experiments of FLOPs and parameters, this module will not increase too many parameters and maintain a fast speed. From the comparative experiments, MCAM uses the network to obtain the best results on the whole-brain segmentation task with multi-grained features and improves the Dice coefficient by about 1.5% compared with FastSurfer on the JHU dataset. From the ablation experiments, when the attention contexts of different branches are different, the improvement effect is more obvious. This effectively shows that the different size convolution kernels of each branch help to improve the recognition effect of multi-grained information.

According to the ablation experiments, the complex backbone network cannot improve the accuracy of the model, because the complex detailed information might not exist in the high-level semantic information, but in the low-level semantic information. According to the ablation experiments, the start part of ResNet and VGG networks lacks rich convolutional layers, whereas the initial part of ResNet is a single-layer convolutional layer and max-pooling, and VGG contains just 2 convolutional layers. They lose a lot of low-level semantic information, and our residual dense block is composed of three groups of convolutional groups, so the low-level semantic information is well maintained by the encoder block. The final results show that MRF-Net +decoder (a) (w/o REFM) improves the Dice coefficient by about 0.7% in the JHU dataset compared to other backbone networks.

In addition to the above comparisons, we conducted a reliability analysis and reported ICC and PCC, these two parameters show that the segmentation results of MRF-Net are closer to the ground truth. In addition, we list some ICC values of important brain regions. It can be seen that our results are significantly better than FastSurfer. This might be caused by the optimized multiple structures and fuzzy boundaries by MCAM and REFM, respectively.

Our MRF-Net is trained independently and can be easily appended to any existing whole-brain maps and researchers could easily build themselves' segmentation models. We hope that this work will contribute to large-scale brain science research. In future study, we will explore the clinical feasibility of this method and verify its performance in other cases with serious anatomical atrophy such as Alzheimer's Disease.

## Data availability statement

The original contributions presented in the study are included in the article/supplementary material. The source code of MRF-Net is available on https://github.com/weichonghit/brain_seg.git. Further inquiries can be directed to the corresponding author.

## Ethics statement

Ethical review and approval was not required for the study on human participants in accordance with the local legislation and institutional requirements. The patients/participants provided their written informed consent to participate in this study.

## Author contributions

CW: methodology, project administration, and writing the draft. YY: methodology and writing-review and editing. XG: data clean and preprocessing. CY and YX: review and editing. HL: data collection and investigation. TM: project administration, supervision, and funding acquisition. All authors contributed to the article and approved the submitted version.

## Funding

The study is supported by grants from the Innovation Team and Talents Cultivation Program of the National Administration of Traditional Chinese Medicine (ZYYCXTD-C-202004), Shenzhen Longgang District Science and Technology Development Fund Project (LGKCXGZX2020002), Basic Research Foundation of Shenzhen Science and Technology Stable Support Program (GXWD20201230155427003–20200822115709001), the National Key Research and Development Program of China (2021YFC2501202), and the National Natural Science Foundation of China (62106113).

## Conflict of interest

Author HL was employed by MindsGo Co., Ltd. The remaining authors declare that the research was conducted in the absence of any commercial or financial relationships that could be construed as a potential conflict of interest.

## Publisher's note

All claims expressed in this article are solely those of the authors and do not necessarily represent those of their affiliated organizations, or those of the publisher, the editors and the reviewers. Any product that may be evaluated in this article, or claim that may be made by its manufacturer, is not guaranteed or endorsed by the publisher.
